# 

**Published:** 1952-01

**Authors:** 


### THE BRISTOL

### MEDICO-CHIRURGICAL JOURNAL

*A Journal of the Medical Sciences for the*

### WEST OF ENGLAND AND SOUTH WALES

PUBLISHED UNDER THE AUSPICES OF THE
BRISTOL MEDICO-CHIRURGICAL SOCIETY

*Editor:*

H. KEITH LUCAS
M.CH., ORTH., F.R.C.S.

*with whom are associated*

J. E. CATES, M.D., M.R.C.P., A. H. GALE, M.D., D.P.H.: *Assistant Editors*
G. E. F. SUTTON, *M.C.*, M.D., F.R.C.P.
J. H. MIDDLEMISS, M.D., D.M.R.D.
A. M. MacLACHLAN, M.B., CH.B.
F. J. Y. WOOD, M.D., M.R.C.P.

*"Scire est nescire, nisi id me
Scire alius sciret."*

*1952 Vol. 69*

J. W. ARROWSMITH LTD., SMALL STREET, BRISTOL

WI
BR323

### *1952 Contents of Vol. 69*

*page*

Eczema of the Colon: a New Conception of Ulcerative Colitis. By JOHN NAISH, M.D., M.R.C.P. . . . . . . . . . . 5

Maternity Service in Bristol: Some Aspects of Integration and Possible Development. By G. GORDON LENNON, CH.M., M.R.C.O.G., M.M.S.A. . . . 10

The Medical Witness for the Prosecution and the Defence. By FRANCIS E. CAMPS, M.D. . . . . . . . . . . 13

Poliomyelitis in Bristol, 1950. By JAMES MACRAE, M.D., F.R.F.P.S., D.P.H. . 20

The Prevention and Treatment of Postpartum Haemorrhage in Domiciliary Practice. By DAME LOUISE McILROY, M.D., F.R.C.P., F.R.C.O.G. . . . 35

The Symptoms and Clinical Diagnosis of Hiatus Hernia. By RONALD BELSEY, M.S., F.R.C.S. . . . . . . . . . . 39

Contact Dermatitis. By CLIFFORD D. EVANS, O.B.E., M.B., B.CH. . . 45

Bristol's New Health Centre in Leinster Avenue, Knowle West. By R. C. WOFINDEN, M.D., D.P.H. . . . . . . . 49

On Reactions with the Newer Antibiotics. By P. G. DALGLEISH, M.D., M.R.C.P. 54

Views Ancient and Modern on Placents Praevia. By JOHN STALLWORTHY, F.R.C.S., F.R.C.O.G. . . . . . . . . . 57

Unexplained Pyrexia in a Child, from Carcinoma of the Adrenal Cortex . 60

The Diagnosis of Abdominal Emergencies in Children. By R. E. HORTON, M.B.E., F.R.C.S. . . . . . . . . . . 77

Unexplained Increase in Fatal Pulmonary Embolus at the Bristol Royal Infirmary in 1950 . . . . . . . . . 82

Gargoylism. By C. R. CROFT, D.M., M.R.C.P. . . . . . . 90

The Relation Between Hypertension and the Kidney: a Review. By HARRY SOSNOWICK, B.SC., M.B., CH.B. . . . . . . 93

The Surgical Treatment of Bronchiectasis. By J. L. GRIFFITH, F.R.C.S. . . 98

Unilateral Papillary Necrosis of Kidney in Diabetes: a rare complication of enlargement of the prostate gland . . . . . . . . 100

Visit of Dr. Alfred Blalock . . . . . . . 102

Life for the Premature Baby. By BERYL CORNER, M.D. . . . . 117

CONTENTS

| | page |
|---|---|
| Recent Trends in Blood Transfusion and Resuscitation. By GEOFFREY H. TOVEY, M.D. | 125 |
| Tuberculous Meningitis | 130 |
| The Results in 178 Cases of Prefrontal Leucotomy at the Bristol Mental Hospitals | 134 |
| The William Budd Health Centre | 141 |
| Argyria from the Use of Nasal Drops | 143 |
| EDITORIAL NOTES | 3, 33, 75 |
| ANNOTATIONS | 23, 69, 109 |
| REPORTS OF MEETINGS | 25, 64, 104, 145 |
| CORRESPONDENCE | 25, 71, 110 |
| APPOINTMENTS | 30, 73, 112, 148 |
| NOTES AND NEWS | 31, 74, 114, 151 |

### 1952 Index to Vol. 69

Abdominal Emergencies, the Diagnosis of, in Children.—R. E. Horton, 77.

Aims and Limitations of Insulin Therapy. —W. Oakley, 145

Ambulance Service, Reorganization of the, 23

Anaesthesia, What is New in.—E. J. Powell, 66

Argyria from the Use of Nasal Drops, 143

Baby, Premature, Life for the.—B. Corner, 117

Belsey, R.—The Symptoms and Clinical Diagnosis of Hiatus Hernia, 39

Binnie, M. C.—Problems of Geriatrics, 66

Blalock, Dr. Alfred, Visit of, 102

Blood Transfusion and Resuscitation, Recent Trends in.—G. H. Tovey, 125

Bristol's New Health Centre in Leinster Avenue, Knowle West.—R. C. Wofinden, 49

Bronchiectasis, the Surgical Treatment of.—J. L. Griffith, 98

Camps, F. E.—The Medical Witness for the Prosecution and the Defence, 13

Clinical Cardiography, Scope of.—G. M. Colson, 65

Colson, G. M.—The Scope of Clinical Cardiography, 65

Common Out-Patients Problems.—W. A. Lister, 146

Contact Dermatitis.—C. D. Evans, 45

Corner, B.—Life for the Premature Baby, 117

Croft, C. R.—Gargoylism, 90

Dalgleish, P. G.—On Reactions with the Newer Antibiotics, 54

Defence of the Air Passages: Second Patrick Watson-Williams Memorial Lecture.—V. E. Negus, 68

Diagnosis of Abdominal Emergencies in Children, The.—R. E. Horton, 77

Eczema of the Colon.—J. Naish, 5

Evans, C. D.—Contact Dermatitis, 45

Gargoylism.—C. R. Croft, 90

Griffith, J. L.—The Surgical Treatment of Bronchiectasis, 98

Hare, E. H.—The Results in 178 Cases of Prefrontal Leucotomy at the Bristol Mental Hospitals, 134

Hiatus Hernia, the Symptoms and Clinical Diagnosis of.—R. Belsey, 39

Hinde, R.—Tonsils and Adenoids, 64

Home Nursing Equipment, 23

Horton, R. E.—The Diagnosis of Abdominal Emergencies in Children, 77

Hypertension and the Kidney, the Relation Between.—H. Sosnowick, 93

Insulin Therapy, Aims and Limitations of.—W. Oakley, 145

Kidney, the Relation Between Hypertension and the.—H. Sosnowick, 93

Kidney, Unilateral Papillary Necrosis of, in a Diabetic, 100

Lennon, G. G.—Maternity Services in Bristol, 10

Life for the Premature Baby.—B. Corner, 117

Lister, W. A.—Common Out-Patient Problems, 146

Macrae, J.—Poliomyelitis in Bristol, 20

Maternity Service in Bristol.—G. G. Lennon, 10

McIlroy, Dame Louise.—The Prevention and Treatment of Postpartum Haemorrhage in Domiciliary Practice, 35

McKendrick, G. D. W.—Tuberculous Meningitis, 130

Medical Hazards Confronting the Modern Aviator.—L. S. White, 64

Medical Witness for the Prosecution and the Defence, The.—F. E. Camps, 13

Meningitis, Tuberculous.—G. D. W. McKendrick, 130

Naish, J.—Eczema of the Colon, 5

Nasal Drops, Argyria from the Use of, 143

Necrosis of Kidney, Unilateral Papillary, in a Diabetic, 100

Negus, V. E.—Second Patrick Watson-Williams Memorial Lecture: Defence of the Air Passages, 68

Newer Antibiotics, On Reactions with the.—P. G. Dalgleish, 54

Oakley, W.—Aims and Limitations of Insulin Therapy, 145

Placenta Praevia, Views Ancient and Modern on.—J. Stallworthy, 57

Poliomyelitis in Bristol, 1950.—J. Macrae, 20

Postpartum Haemorrhage, the Prevention and Treatment of, in Domiciliary Practice.—Dame Louise McIlroy, 35

Powell, E. J.—What is New in Anaesthesia, 66

Prefrontal Leucotomy, the Results in 178 Cases of, at the Bristol Mental Hospitals.—E. H. Hare, 134

Premature Baby, Life for the.—B. Corner, 117

Problems of Geriatrics.—M. C. Binnie, 66

Pulmonary Embolus, Unexplained Increase of Fatal, at the Bristol Royal Infirmary in 1950.—M. G. Wilson, 82

Pyrexia, Unexplained, in a Child, 60

Reactions with the Newer Antibiotics, On.—P. G. Dalgleish, 54

Recent Trends in Blood Transfusion and Resuscitation.—G. H. Tovey, 125

Relation Between Hypertension and the Kidney.—H. Sosnowick, 93

Reorganization of the Ambulance Service, 23

Results in 178 Cases of Prefrontal Leucotomy at the Bristol Mental Hospitals.—E. H. Hare, 134

Scope of Clinical Electrocardiography.—G. M. Colson, 65

Sosnowick, H.—The Relation Between Hypertension and the Kidney, 93

Stallworthy, J.—Views Ancient and Modern on Placentia Praevia, 57

Surgical Treatment of Bronchiectasis, The.—J. L. Griffith, 98

Tonsils and Adenoids.—R. Hinde, 64

Tovey, G. H.—Recent Trends in Blood Transfusion and Resuscitation, 125

Tuberculous Meningitis.—G. D. W. McKendrick, 130

Unexplained Pyrexia in a Child, 60

Unilateral Papillary Necrosis of Kidney in a Diabetic, 100

Views Ancient and Modern on Placenta Praevia.—J. Stallworthy, 57

White, L. S.—The Medical Hazards Confronting the Modern Aviator, 64

William Budd Health Centre, The, 141

Wilson, M. G.—Unexplained Increase of Fatal Pulmonary Embolus at the Bristol Royal Infirmary in 1950, 82

Wofinden, R. C.—Bristol's New Health Centre in Leinster Avenue, Knowle West, 49

THE BRISTOL

MEDICO-CHIRURGICAL JOURNAL

*A Journal of the Medical Sciences for the*

WEST OF ENGLAND AND SOUTH WALES

VOL. 69 JANUARY 1952 NO. 249

*PUBLISHED QUARTERLY*

*ANNUAL SUBSCRIPTION 16/- POST FREE*

PUBLISHED UNDER THE AUSPICES OF THE
BRISTOL MEDICO-CHIRURGICAL SOCIETY

### *Editorial Committee*

MR. H. KEITH LUCAS, 60 St. John's Road, Bristol 8. *Editor.*
DR. J. E. CATES, The Dept. of Medicine, Bristol Royal Infirmary. *Assistant Editor.*
DR. G. E. F. Sutton, 1 Pembroke Road, Bristol 8. *Treasurer.*
DR. J. H. MIDDLEMISS, 11 Pembroke Road, Bristol 8.
DR. A. M. MACLACHLAN, 167 Redland Road, Bristol 6.
DR. A. H. GALE, The University, Bristol 8.

*Local Correspondents*

*Bath* . . Mr. A. D. BATEMAN, 3 The Circus, Bath.
*Exeter* . . Dr. L. N. JACKSON, Mount Jocelyn, Crediton, Devon.
*Gloucester*. Dr. R. F. JARRETT, 9 College Green, Gloucester.
*Newport* . Mr. J. L. D. WILLIAMS, The Knoll, 145 Stow Hill, Newport, Mon.
*Plymouth* . Dr. C. R. CROFT, 1 The Crescent, Hartley, near Plymouth.
*Taunton* . Dr. M. MCALLEY, 1 The Crescent, Taunton.
*Torquay* . Dr. A. ROBINSON THOMAS, 20 Devon Square, Newton Abbot, Devon.
*Truro* . . Dr. F. D. M. HOCKING, St. Andrews, Perranporth, Cornwall.
*Yeovil* . . Mr. J. A. VERE NICHOLL, Knapp House, Yeovil, Somerset.

### NOTICE TO CONTRIBUTORS

Original articles are invited, provided they have not been published elsewhere. For the present we are, unfortunately, compelled to fix a limit of two thousand words.

Notes of forthcoming events, meetings held, degrees conferred, staff appointments, and other local news are welcomed.

Illustrations should always be supplied ready for reproduction. All re-touchings or other operations required will be charged to the author. Line drawings should be drawn in Indian ink. We regret that we must ask authors to pay for making blocks, if this is necessary.

Authors of original articles are entitled to receive twenty-five Reprints, with covers showing source of Reprint, free of charge. Additional reprints may be obtained if notice is given at the time of returning proofs. Approximate cost, with cover as above: 8 pp., 25 copies, 8/-; 50 copies, 12/6. Contributors requiring special covers for their reprints, showing source of reprint with author's name, appointments and title of article, can obtain them as follows: 25 copies, 12/-; 50 copies, 14/6.

J. W. ARROWSMITH LTD., SMALL STREET,
BRISTOL.

### *Appointments*

*Bath and Bristol*

ADAMS, A. K., D.A.: Registrar in Anaesthetics, Frenchay Hospital.

CLARKE, SUZANNE K. R., M.B., B.Ch.: Registrar in the Pathological Department, United Bristol Hospitals.

CREE, G. E., M.R.C.S., L.R.C.P.: Assistant Venereologist, Bristol.

DAVIES, D. T., M.R.C.S., L.R.C.P.: Assistant Physician in Chest Diseases, Bristol.

FINCH, D. C., M.D.: Registrar in Diseases of the Chest, Frenchay Hospital.

HARE, E. H., D.P.M., M.D.: Assistant Psychiatrist, Bristol.

HENWOOD, A. R., B.Ch.D.: Assistant Dental Surgeon, Bath.

KERR, A. R., M.B., Ch.B.: Registrar in Anaesthetics, Southmead Hospital.

LAFFERTY, E. M., D.A.: Anaesthetist, Bristol.

LAWSON, J. B., D.(Obst.)R.C.O.G.: Registrar in Obstetrics and Gynaecology, Southmead Hospital.

LEWIS, W. J., D.A.: Assistant Anaesthetist, Bath.

REED, B. O., M.B., B.Ch., M.R.C.S., L.R.C.P.: Principal in General Practice, Bristol Executive Council.

REES, R. E. L., D.O.M.S.: Ophthalmic Registrar, Eye Hospital.

ROBINSON, H. H., D.M.R.T.: Assistant Radiotherapist, United Bristol Hospitals.

ROSS, D. N., F.R.C.S.: Senior Registrar in Thoracic Surgery, Frenchay Hospital.

ROUTLEDGE, R. T., F.R.C.S.: Senior Registrar in Plastic Surgery, Frenchay Hospital.

SHEACH, JEAN M., D.M.R.D.: Assistant Radiologist, United Bristol Hospitals.

SHIELDS, D. W., M.R.C.O.G.: Registrar in Obstetrics and Gynaecology, United Bristol Hospitals.

SMITH, S., M.D., D.P.M.: Consultant Psychiatrist, Bristol.

TOOMEY, A. A. G., D.M.R.D.: Registrar in the Radiological Department, United Bristol Hospitals.

TROTTER, J. K., M.R.C.S., L.R.C.P.: Registrar in Anaesthetics, Frenchay Hospital.

TRUSCOTT, D. E., D.M.R.D.: Radiological Registrar, United Bristol Hospitals.

TWOMEY, J. G., M.B., B.Ch., B.A.O., N.U.I.: Principal in General Practice, Bristol Executive Council.

WATSON, P. C., F.R.C.S.: Senior Registrar in General Surgery, Southmead Hospital.

*Bristol Mental Hospital, Fishponds.*

Registrars in Psychiatry.
BOWEN, L. W., M.B., B.S.
EDGAR, B., M.B., Ch.B.
FRENCH, A., M.B., Ch.B.
KAUFMAN, H., M.R.C.S., L.R.C.P.
MILNE, C., M.B., D.P.M.

*Devizes*

ARMIN, R. H., D.P.M.: Assistant Psychiatrist, Roundway Hospital.

*Exeter*

MERRYWEATHER, R., F.R.C.S.: Senior Registrar in Orthopaedics, Princess Elizabeth Orthopaedic Hospital.

ROBINS, R. H. C., F.R.C.S.: Registrar in Orthopaedics, Princess Elizabeth Orthopaedic Hospital.

*Gloucester*

JAMES, P. A., F.R.C.S.: Registrar in General Surgery, Gloucestershire Royal Infirmary.

*Plymouth*

HARDING-COX, J., M.B., Ch.B.: Registrar in Orthopaedics, Mount Gold Orthopaedic Hospital.

WALDRON, E. A., D.M.R.D.: Consultant Radiologist.

*South Somerset*

LLEWELYN JONES, T., M.B., Ch.B.: Assistant Anaesthetist.

*Taunton*

RUSSELL, HELEN M., M.R.C.O.G.: Senior Registrar in Obstetrics and Gynaecology, Taunton and West Somerset Hospital.

*Wells*

BRIDGER, W. E. W., D.P.M., M.D.: Consultant Psychiatrist, Mendip Hospital.

*West Cornwall*

DUTTON, JEAN, D.A.: Assistant Anaesthetist.

FELDMESSER, E. E., M.D.: Assistant Psychiatrist, St. Lawrence's Hospital, Bodmin.

